# Proposal of requirements for the development of a training simulator for the auditory-perceptual judgment of voice

**DOI:** 10.1590/2317-1782/20232022209en

**Published:** 2023-10-06

**Authors:** Maxsuel Alves Avelino de Paiva, Liliane dos Santos Machado, Leonardo Wanderley Lopes

**Affiliations:** 1 Universidade Federal da Paraíba - UFPB, João Pessoa (PB), Brasil.

**Keywords:** Voice, Vocal Quality, Auditory Perception, Training, Simulation

## Abstract

**Purpose:**

to identify a set of requirements for the development of an auditory-perceptual training simulator (APT) based on the experience of professors who provide APT.

**Methods:**

This is a cross-sectional, descriptive study with a quantitative approach. Twenty-two professors answered an online questionnaire containing 31 items related to APT, involving items about the professional profile, conditions for APT in undergraduate and postgraduate courses in Speech Therapy, APT structure, and evaluation of the APT effect.

**Result:**

it was observed that there is a variation in APT procedures performed in Brazil. The main requirements indicated by the respondents for the APT involve the use of synthesized voices in the initial moments, followed by human voices later; the use of speech tasks with sustained vowels and connected speech; the insertion of complementary information such as gender, age, the profession of the speaker and the spectrography of the vocal signal; training with a minimum time of six hours; the evaluation of the training effect by comparing intra- and inter-judge agreement before and after training; the addition of the parameters of general degree of vocal deviation, roughness, breathiness, and strain; the use of validated continuous and numerical scales; and offering it from the second year of the undergraduate program.

**Conclusion:**

although there is variability in the response of experts, a minimum set of requirements indicated for performing APT with new judges was identified.

## INTRODUCTION

Auditory-perceptual evaluation (APE) of voice is considered the gold-standard method used by speech-language-hearing (SLH) therapists in clinical voice assessment. In it, the evaluator judges, based on their auditory impression (perception), the speaker’s voice characteristics, such as voice quality, pitch, loudness, resonance, articulation, and so on^(1)^. APE identifies the presence/absence of vocal deviations, characterizes the intensity and type of vocal deviation, and provides important information on the social acceptability of that voice. Since it is based on impressions, APE is subjective and influenced by various factors, including the judge’s training^(2)^. The subjectivity and arbitrariness inherent to this method may justify the tendency to name it “auditory-perceptual judgment” (APJ), rather than APE^(3,4)^.

Training APJ skills changes the perception system and auditory information processing, improving the listener’s capacity to respond to stimuli to which they have been trained. Such changes create an auditory memory that is accessed in future assessments, enabling them to recognize patterns deemed normal or deviated^(5)^.

Strategies such as auditory anchors, immediate feedback, and standardized scales are used in auditory-perceptual training (APT) to minimize its subjectivity^(6-9)^. These strategies have negative aspects, such as expenses with material (paper, pen, loudspeaker, and so forth), the unfeasibility of immediately analyzing the effects of training and the availability of judges to in-person meetings. Moreover, APT is conducted in various ways, hindering the comparison of training requirements and effects^(10)^.

New technologies are being used to complement traditional teaching strategies with interactive approaches. Applications such as training simulators (TS) and serious games provide controlled experiences, including various situations professionals will find in real scenarios, helping them learn and transfer such skills to practical work^(11)^. Virtual environments allow for making mistakes and correcting them from the initial phases of training without any consequences to either patients or students. The user’s performance can also be immediately assessed with objective measures obtained from their interaction with the virtual environment^(11)^.

The first stage to develop an application for this type of training is defining the training structure, requirements, and parameters that will be used in the application and then assessing the users’ performance^(11)^. The lack of well-established such definitions in the literature and/or consensus between researchers in the area^(12)^ poses a challenge to proposing a TS. In this case, combining specialists’ practical experience and the knowledge reported in the literature is the main strategy to define requirements and model a robust TS adequate to real needs^(13)^.

There may be occasional divergences between the knowledge available in the literature and the training that takes place in specialists’ everyday practice. Moreover, some requirements pointed out in the literature are subjective and need to be more clearly and objectively translated for implementation in TS. Studies approaching APT^(10,14)^ are not consistent in terms of training time, number of stimuli and vocal parameters, type of scale, and nature of the voices they use. Above all, they do not indicate a hierarchy to present stimuli and tasks in APT (such as training the presence/absence of vocal deviations, vocal deviation predominance, and degrees of vocal deviation presented in a sequence). This justifies consulting professors specialized in voice and experienced in APT to establish requirements to bridge these gaps and support TS modeling definitions for APT.

Establishing APT requirements may help develop a TS for this assessment. An APT simulator modality may help structure and standardize training, analyze performance, understand the judge’s learning curve, and flexibilize the training time. It can also be carried out in settings other than institutions, as no in-person meetings are needed to train with the simulator. Thus, this research aimed to identify a set of requirements to develop a TS for APT based on the experience of professors who provide APT.

## METHODS

This quantitative, descriptive, cross-sectional study was approved by the originating institution’s Research Ethics Committee under evaluation report no. 4.746.039 and conducted between April and July 2021.

Firstly, researchers consulted recently published reviews on APJ and APT^(10,14)^. They found inconsistencies in the training processes concerning training time, number of stimuli and vocal parameters, types of scale, and nature of the voices they approached. Hence, consulting specialists in voice that provide APT aims to minimize these inconsistencies and ground the definitions to model a TS for APT.

The questionnaire used in this research was developed in three stages to ensure the instrument would support the definition of requirements to develop a TS for APT, namely: consulting the literature and surveying the specialists’ opinions on APT and the development of simulation tools. In the first questionnaire development stage, the literature available was surveyed to identify the main variables to be addressed in APT^(10,14)^. The following variables were included in this stage: associated information during APT; number of hours; types of voices; number of voices; types of speech tasks; scales; and parameters.

In the second stage, the variables selected in the literature survey were presented to an SLH therapist who specialized in voice and a computer scientist, both experienced in developing simulation tools to train health professionals’ skills. The questions in this stage were structured according to the questionnaire model developed by Macedo and Machado (2015), who researched, along with professors, the requirements to train health professionals to inject medications. This model was adapted due to the lack of instruments in the field of voice aimed at understanding the requirements for APT. Thus, the authors of the said publication formalized a script to develop instruments to obtain information from specialists to define requirements for simulators.

The process of developing and implementing a TS requires interdisciplinary professional work to produce effective and efficient tools. Computer scientists work in TS arts, entertainment, artificial intelligence, and programming. Hence, they are part of interdisciplinary teams that develop applications, working along with expert professionals in the area to which the product is intended^(11)^. The participation of a computer scientist in this project was likewise essential to TS programming, artificial intelligence, and graphic design. She also participated in the questionnaire conceptualization, structuring the questions to include relevant variables to be selected, as well as other important ones to TS modeling, such as year/period of APT; prerequisites to begin APT; auditory description of the parameters that are trained; hierarchy of APT stages; other applications (games or simulators) used in APT; and form of APT effect assessment.

Three SLH therapists specialized in voice with expertise in APT participated in the third stage, through snowball sampling (a recruited specialist indicated another one). The first SLH therapist works in the originating institution and belongs to the same research group as the authors, though not participating in the previous stages. The second one works in the same institution as the researchers, but not in that research group. The third one is not from the originating institution. In this stage, the specialists’ expertise was assessed with the Fehring Model, with scores adapted to the area of voice^(15)^ (APT), in which the three specialists obtained the maximum score.

The questionnaire developed in the second stage (31-item version) was individually and remotely (via video call) presented to the three SLH therapists to identify and change items that were not coherent with their intended collection and the respondent’s interpretation. The specialists were asked what they had understood after reading each item. If their interpretation was not aligned with the intended collection, the item had to be reformulated based on the participating specialists’ suggestions. No misalignments were identified in this stage between the questionnaire and the specialists’ interpretations. However, they made some suggestions to improve its quality, leading to the following changes: text adjustments (e.g., from “Do you use other resources in APT?” to “In APT, do you associate any other information regarding the voice that is presented?”); changes in the type of response collection (e.g., using “both” instead of “human” and “synthesized” among the multiple-choice answers to the item “The voice used in training are:”, thus making posterior analysis easier); and reducing the number of open-ended questions to decrease the time taken to answer the questionnaire and facilitate professors’ participation, with a more practical instrument, as in the item “What speech task do you use in APT with CAPE-V?”.

The final version of the questionnaire had 31 items (five open-ended and 26 closed-ended questions), organized into four blocks: 1) Professional profile; 2) Conditions for APT in undergraduate and postgraduate SLH programs; 3) APT structure; and 4) APT effect assessment. It was divided into blocks to understand the specific topic addressed by each item and make it easier to analyze and discuss results. The division was made by the authors and approved by the specialist SLH therapists.

To recruit participants, e-mails were sent to the coordinators of 81 undergraduate SLH programs and five postgraduate specialization programs in voice, requesting the contact of the professors responsible for the APT of undergraduate SLH students or postgraduate SLH therapists. This research was also presented directly to some such professors. The e-mail has a brief description of the research, its objectives, participation criteria, and a link to the informed consent form.

The following eligibility criteria were established for this research, considering its objective: being an undergraduate or postgraduate SLH professor; having experience in teaching any course that includes APT; having conducted APT at least once. Participants that met these criteria and agreed with the informed consent form were invited to continue, answering the questionnaire in Google Forms.

Hence, the final sample had 22 professors, of which 15 (68.2%) taught in undergraduate and master’s programs, two (9.1%) taught only in specialization programs in voice, and five (22.7%) trained undergraduate and specialization students in voice. Considering that one professor per institution provides APT, the sample comprised about 26% of the population. Despite the efforts to have more professors participate in the research, many e-mails were not answered, even after sending them three times.

The sample had representatives from three regions of Brazil (Northeast, Southeast, and Central-West), including the following federative units: São Paulo (n = 10, 45.5%), Pernambuco (n = 3, 13.6%), Paraíba (n = 3, 13.6%), Minas Gerais (n = 2, 9.1%), Rio de Janeiro (n = 2, 9.1%), Federal District (n = 1, 4.5%), and Rio Grande do Norte (n = 1, 4.5%). According to their professional profile, most interviewees (n = 13, 59.1%) had a doctoral degree and had been teaching for more than 10 years in undergraduate programs at public institutions, where they provide APT in required courses.

The data spreadsheet was extracted from Google Forms to calculate the relative frequency measures of closed-ended items. The open-ended items were qualitatively analyzed and grouped into categories according to the content of the answers.

## RESULTS

Concerning the conditions for APT, most interviewees (n = 21, 95.5%) reported that it is offered to undergraduate students in or after the second year of the SLH program. Also, 15 interviewees (68.18%) stated that there is a better moment during the undergraduate program to provide APT, and all of them agree that it is after its second year (Chart 1).

**Chart 1:** Questionnaire items and participants’ responses

BLOCK 1 - PROFESSIONAL PROFILE		
ITEMS		***INTERVIEWEES’ RESPONSES***
1	What is your highest academic degree?	*Doctoral (n = 13, 59,1%)*
		*Postdoctoral (n = 9, 40.9%)*
2*	What is the target public of your auditory-perceptual training of voice?	*Undergraduate degree (n = 19, 86.4%)*
		*Specialization in voice (n = 7, 31.8%)*
		*Master’s degree (n = 9, 40.9%)*
		*Extension courses (n = 5, 22.7%)*
		*Residence (n = 1, 4.5%)*
3	If you teach undergraduate courses, is auditory-perceptual training to assess the voice quality part of the content in any required course?	*Yes (n = 15,68.2%)*
		*No (n = 1, 4.5%)*
		*NA (n = 6, 27.3%)*
4*	In what type of institution do you teach?	*Public (n = 18, 81.9%)*
		*Private (n = 4, 18.2%)*
		*Foundation (n = 1, 4.5%)*
5	In what federative unit is the institution located?	*São Paulo (n = 10, 45.5%), Paraíba (n = 3, 13.6%), Pernambuco (n = 3, 13.6%), Minas Gerais (n = 2, 9.1%), Rio de Janeiro (n = 2, 9.1%), Federal District (n = 1, 9.1%), and Rio Grande do Norte (n = 1, 9.1%)*
6	For how long have you been teaching?	*More than 10 years (n = 18, 81.1%)*
		*Less than 10 years (n = 4, 18.2%)*
BLOCK 2 - CONDITIONS FOR AUDITORY-PERCEPTUAL TRAINING		
ITEMS		***INTERVIEWEES’ RESPONSES***
1	In what year of their undergraduate studies do students receive auditory-perceptual training at the institution where you teach?	*From the 2^nd^ year on (n = 21, 95.5%)*
2	Do you believe there is a better moment throughout the undergraduate program to provide auditory-perceptual training to speech-language-hearing students?	*Yes (n = 15, 68.2%)*
		*All of them answered it should take place from the second year on*
		*No (n = 7, 31.8%)*
3	Is there any prerequisite for students to begin this training at the institution where you teach?	*Yes (n = 10, 45.5%)*
		*No (n = 12, 54.5%)*
BLOCK 3 - STRUCTURE OF THE AUDITORY-PERCEPTUAL TRAINING		
ITEMS		***INTERVIEWEES’ RESPONSES***
1*	Do you associate any other information regarding the voices presented in auditory-perceptual training?	*Age (n = 18, 81.1%)*
		*Sex (n = 18, 81.1%)*
		*Main complaint (n = 14, 63.6%)*
		*Spectrogram (n = 11, 50%)*
		*Laryngeal examination (n = 10, 45.4%)*
2	How many total hours are there in classroom training?	*Less than 2 hours (n = 2, 9.1%)*
		*From 2 to 4 hours (n = 5, 22.7%)*
		*From 4 to 6 hours (n = 3, 13.6%)*
		*From 6 to 8 hours (n = 5, 22.7%)*
		*More than 8 hours (n = 4, 18.1%)*
		*Other (n = 3, 13.6%)*
3	Are the voices used in training human or synthesized?	*Humans (n = 17, 77.3%)*
		*Human and Synthesized (n = 5, 22.7%)*
4	How many voices do you use in training?	*Up to 20 voices (n = 8, 36.4%)*
		*From 21 to 40 voices (n = 7, 31.8%)*
		*More than 50 voices (n = 7, 31.8%)*
5*	Which speech tasks do you use in training?	*Number count (n = 21, 95.5%)*
		*CAPE-V sentences (n = 17, 77.3%)*
		*Spontaneous speech (n = 17, 77.3%)*
		*Vowel “a” (n = 15, 68.2%)*
		*Vowel “é” (n = 12, 54.5%)*
6	Do you use CAPE-V (Kempster et al.^(16)^) in auditory-perceptual training?	*Yes (n = 18, 81.8%)*
		*No (n = 4, 18.2%)*
7	Do you use GRBAS (Hirano^(17)^) in auditory-perceptual training?	*Yes (n = 20, 90.9%)*
		*No (n = 2, 9.1%)*
8	Do you use the Vocal Deviation Scale - VDS (Yamasaki et al.^(18)^) in auditory-perceptual training?	*Yes (n = 13, 59.1%)*
		*No (n = 9, 40.9%)*
9**	Suppose there are 3 hierarchical levels of complexity in auditory-perceptual training. Please, list them in the order you would use them in training.	*1^st^ Level: Identifying the presence of vocal deviation (n = 20, 90.9%)*
		*2^nd^ Level: Assessing the predominating vocal quality (n = 15, 68.2%)*
		*3^rd^ Level: Assessing the general degree of vocal deviation (n = 12, 54.5%)*
10*	Which parameters do you address in training?	*Roughness (n = 22, 100%)*
		*Breathiness (n = 22, 100%)*
		*Strain (n = 22, 100%)*
11^#^	How do you define to students the auditory characteristics expected from a rough voice?	*Nonspecific responses when defining auditory characteristics or defining them with physiological/anatomical correlates:*
		*“Irregular vibration”, “noise”, “dirty voice”, “sandy voice”*
12^#^	How do you define to students the auditory characteristics expected from a breathy voice?	*The responses mentioned “Any audible air escape during voice production”*
13^#^	How do you define to students the auditory characteristics expected from a strained voice?	*Nonspecific responses when defining auditory characteristics or defining them with physiological/anatomical correlates:*
		*“Tight sensation”, “vocal effort”, “vocal hyperfunction”*
14	How many hours of training would you consider enough to improve students’ performance (rate of correct answers) and reliability in auditory-perceptual evaluation?	*Up to 4 hours (n = 3, 13.6%)*
		*From 5 to 8 hours (n = 5, 22.7%)*
		*More than 8 hours (n = 14, 63.3%)*
15	Do you use any type of game or simulator in auditory-perceptual training?	*Yes (n = 1, 4.5%)*
		*No (n = 21, 95.5%)*
BLOCK 4 - ASSESSMENT OF AUDITORY-PERCEPTUAL TRAINING EFFECTS		
ITEMS		***INTERVIEWEES’ RESPONSES***
1	*Do you assess students’ reliability in auditory-perceptual evaluation after auditory-perceptual training?*	Yes (n = 9, 40.9%)
		No (n = 13, 59.1%)
2#	*How do you assess the effects of auditory-perceptual training on the students’ performance and reliability?*	The responses mention: “Interrater and intrarater agreement tests; observing and discussing evaluations; a formal test”
3	*Do you have difficulties assessing the effects of auditory-perceptual training? (If you do not assess training effects, check "NA")*	Yes (n = 5, 22.7%)
		No (n = 6, 27.3%)
		Not assessed (n = 11, 50%)
4^#^	*Can you point out any difficulty(ies) in assessing the effects of auditory-perceptual training?*	The difficulties include: “Lack of comparison parameters; lack of calibrating instruments for training; laborious procedures with statistical tests”
5	*How do you define students’ correct answers concerning the general degree of vocal deviation using CAPE-V or VDS?*	When the markings in CAPE-V or VDS coincide with the reference judge’s values or are 10 mm above or below this value (n = 13, 59.1%).
		When the markings in CAPE-V or VDS *coincide with the reference judge’s values or are 5 mm above or below this value (n = 2, 9.1%).*
		When the markings in CAPE-V or VDS *coincide with the reference judge’s values, with no margins of error (n = 2, 9.1%).*
		*I do not use CAPE-V or VDS (n = 4, 18.2%).*
		*Other: (n = 1, 4.5%) “I never used these criteria”.*
6	*How do you assess students’ correct answers when assessing the general degree using GRBAS?*	When they coincide (n = 10, 45.5%)
		When they coincide or is one degree above or below (n = 10, 45.5%)
		I do not use GRBAS (n = 2, 9%)
7	*To what extent do you consider auditory-perceptual training important to speech-language-hearing students’ initial training, on a scale from 0 - (not important) to 5 - (very important)?*	5 (n = 19, 86.4%)
		4 (n = 2, 9.1%)
		3 (n = 1, 4.5%)

**Caption:** * Item in which more than one answer can be checked; ** In this item, the most cited 1^st^, 2^nd^, and 3^rd^ TPA levels are presented; ^#^ Subjective items that allowed professors to come up with their own answers

Respondents generally associate additional information of the speakers in APT, such as their sex (n = 18, 81.8%), age (n = 18, 81.8%), complaint (n = 14, 63.3%), spectrogram (n = 11, 50%), and laryngeal examination result (n = 10, 45.5%).

The total number of APT hours in the classroom range from less than 2 hours to more than 8 hours. Most responses ranged from 6 to 8 hours of training (n = 5, 22.73%).

Most participants use human voices (n = 17, 77.3%) in APT, while five (22.7%) use both human and synthesized voices. The number of voices used in APT is quite evenly distributed among participants into “Up to 20 voices” (n = 8, 36.4%), “21 to 40 voices” (n = 7, 31.8%), and “More than 50 voices” (n = 7, 31.8%).

The most used speech tasks in APT are number count (n = 21, 95.5), spontaneous speech (n = 17, 77.3%), CAPE-V sentences (n = 17, 77.3%), /a/ vowels (n = 15, 68.2%), and /ɛ/ vowels (n = 12, 54.5%). GRBAS (n = 20, 90. 9%) and CAPE-V (n = 18, 81.8%) are the most used scales.

Respondents generally begin APT by identifying the presence/absence of vocal deviation. However, they diverge in the sequence of the subsequent stages. Concerning a possible APT stage hierarchy, most of them gave the following order: identifying the presence of vocal deviation as the first training level (n = 20, 90%), assessing voice quality predominantly as the second level (n = 15, 68.2%), and assessing the degree of vocal deviation as the last level (n = 12, 54.5%).

All interviewees include the general degree of vocal deviation (G), roughness (R), breathiness (B), and strain (S) as APT parameters. They were asked to describe the auditory characteristics related to the training parameters, but they seemed to have difficulties defining those related to R and S. From the auditory standpoint, B seems to be more easily explained, reported by interviewees as “any audible air escape during voice production”. The interviewees’ responses did not specifically define auditory characteristics and/or defined physiological/anatomical correlates of R and S. The responses regarding R referred to “irregular vibration”, “noise”, “dirty voice”, and “sandy voice”. As for S, the responses mentioned “tight sensation”, “vocal effort”, and “vocal hyperfunction”.

More than half of the interviewees (n = 14, 63.3%) consider that APT must last more than 8 hours to improve the judges’ performance and reliability. Most participants (n = 21, 95.5%) do not use any type of simulator or game in APT.

About 60% of interviewees (n = 13) do not assess the judges’ reliability after APT. As for those who assess their reliability after APT, the methods cited are interrater and intrarater agreement tests, observing and discussing assessments, and formal assessment tests. Of these, 22.7% (n = 5) reported difficulties assessing APT effects because of the lack of comparison parameters and calibrating instruments for the training and the unfeasibility of performing statistical test procedures during APT.

More than half of the interviewees (n = 13, 59.1%), who use CAPE-V and the Vocal Deviation Scale (VDS) agree with the assessment that coincides with the reference judge value or is 10 mm above or below this value. As for those who use GRBAS, 50% (n = 10) agree with the assessment that coincides with the degree ascribed to the reference judge, while the other half (n = 10, 50%) admits one degree higher or lower. APT is considered very important to SLH students’ initial training by 86.4% (n = 19) of the interviewees.

## DISCUSSION

APJ is influenced by various factors, including the judges’ training^(2)^. APT models in the literature are inconsistent regarding variables involved in training^(10,14)^. To define the best way of providing APT, we must first know the various training methods that have been used and recorded in the literature. They must also be described to enable an adequate assessment of their results, comparing training methods, and defining to which populations the results may be applicable^(10)^.

It is not an easy task to establish the requirements to provide training through a TS, especially in the case of such subjective training as APT. Hence, the knowledge available in the literature must be combined with specialists’ practical experience to address these difficulties and objectively define the requirements for a robust TS^(13)^.

Thus, it is essential to consult judges experts in APT to plan the training of new SLH therapists and develop training models based on specialists’ opinions^(11)^. Experienced judges have better-defined inner standards and experience to train beginners. Inner standards result from APT and APJ experiences throughout their academic training and career^(12)^. Hence, both professional training (undergraduate and postgraduate formal training) and temporal characteristics (years working with APJ) have been pointed out to determine a judge’s experience^(14)^. This study considered both forms, as all interviewees had a doctoral degree with more than 10 years of teaching and experience in APJ and APT.

This research found that specialists agree that APT must be provided from the second year of undergraduate programs. Initial years’ students take basic courses on health sciences, which are necessary to understand physiological/anatomical behavior regarding the quality of the voices under assessment.

The classification of vocal deviation depends on additional information other than the voice, such as the speaker’s sex, age, and occupation. Moreover, the visual support of the spectrogram tracing can significantly increase voice quality APJ reliability among inexperienced judges, as it increases interrater and intrarater agreement in most analysis parameters^(19)^. These aspects justify adding such information along with the voices used in training inexperienced judges.

Even though most interviewees use human voices in APT, synthesized ones seem to be more adequate for this purpose, especially in the initial moments of the inexperienced judges’ training^(14,20)^. Vocal parameters can be controlled to produce unidimensional synthesized voices (with only one deviated parameter), thus simplifying the inexperienced judges’ assessment^(20)^.

Studies in the area seemingly do not consider the number of voices used in APT as an important variable. It varies considerably, as some studies reported using 30^(8)^, 57^(9)^, and 220^(21)^ voices. Considering APT that encompasses the most universal parameters (R, B, and S), the various degrees (mild, moderate, and intense), and matching per sex (males and females), a range from 30 to 60 voices seems minimally enough to provide APT.

Speech tasks such as sustained vowels and linked speech make it possible to assess both glottal source information and muscle adjustments in the vocal tract^(14)^. Associations between speech tasks, muscle adjustments, and auditory correlates are important to train beginning judges, which justifies the variety of vocal tasks in APT.

The number of hours and stimuli used in APT varied considerably between the interviewees’ responses. These variations are also found in the review of APT methods by Walden and Khayumov (2020), in which the training time ranged from 30 minutes to 20 hours^(10)^. A study^(22)^ used anchor stimuli in APT and found increased intrarater and interrater reliability after 2 hours of training. Given the interviewees’ opinions and studies in the area, the judges’ reliability is expected to increase after 6 to 8 hours of training. A more precise definition of the necessary APT time will only be possible with studies that assess the judges’ performance after different APT training times.

GRBAS and CAPE-V are the most used and accepted instruments worldwide to record APJ in clinical and scientific contexts. These instruments help standardize APE and have particularities in how they are recorded, the parameters they assess, and the type of speech task they use^(14)^. It is not known which one is best to train inexperienced SLH therapists. Hence, the instrument should be chosen based on the training goals, speech samples available, and estimated training time^(14)^.

G, R, B, and S are among the universal parameters most used in APJ^(14)^. G, R, and B have a greater agreement, whereas S has a lower interrater and intrarater agreement and is, therefore, considered less reliable in APJ than the other ones^(14,23,24)^. All interviewees include G, R, B, and S in APT. Thus, including at least these parameters in APE training is justified.

Describing auditory characteristics of R and S poses a challenge to interviewees. Although R is recurrent in clinical voice assessment, interviewees used physiological/anatomical correlates that occur in phonation when trying to describe its auditory characteristics. The same occurred with S, as they mistook effort (speaker’s perception) for strain (muscle contractile activity)^(25)^.

Concerning the sequence of APT levels, identifying the presence/absence of vocal deviations is considered easier for inexperienced judges because it is a categorical, binary classification. On the other hand, characterizing the predominating type of vocal quality deviation requires a more complex categorical classification, including at least three possibilities (roughness, breathiness, and strain). The most complex APT level is believed to be the assessment of vocal deviation intensity, concerning either global deviation or its components (roughness, breathiness, and strain). On this level, assessment is based on a continuum from the absence of a given vocal characteristic to its presence in an intense degree.

One way of assessing APT effects is with intrarater and interrater agreement measures. Statistical agreement tests quantify the proximity of assessments before and after APT^(26)^. Greater proximity is expected between inexperienced judges’ and reference judges’ assessments after APT (interrater agreement). Likewise, inexperienced judges are expected to have more consistent assessments (intrarater agreement). Hence, it is important to assess APT effects to monitor results and implement new necessary strategies.

When using instruments with continuous 100-mm scales in APJ (e.g., continuous CAPE-V and VDS), a variability of up to 10 mm between raters is admissible - as long as such variation does not exceed the limits (cutoff scores) that change the degree of deviatio^(27)^.

## CONCLUSION

The interviewees’ responses varied regarding APT procedures. Given the survey of professors experienced in APJ and the knowledge available in the literature, developing a TS for APT must consider the following requirements: beginning APT with the task of classifying the presence/absence of vocal deviation, advancing to classify the predominating vocal quality, and then classify the degrees of vocal deviation; using synthesized voices in initial moments, progressing later to human voices; using 30 to 60 voices; using speech tasks with sustained vowels and linked speech; adding complementary information, such as the speaker’s sex, age, and occupation and their voice spectrogram; providing at least 6 hours of training; assessing the effects of training by comparing intrarater and interrater agreement before and after training; adding the parameters of general degree of vocal deviation, roughness, breathiness, and strain (at least); using continuous numerical scales; and providing training from the second year of the undergraduate program.

These established requirements are flexible and can be changed as studies advance in the area. However, they are a starting point to propose and develop a TS.
