# Supplementary material for: Proposal of requirements for the development of a training simulator for the auditory-perceptual judgment of voice
Source: Codas. 2023 Oct 6;35(6):e20220209. doi: 10.1590/2317-1782/20232022209en (PMC10702707; doi:10.1590/2317-1782/20232022209en)
Supplement: Translation

# Translation: Proposição de requisitos para o desenvolvimento de um simulador de treinamento para julgamento perceptivo-auditivo da voz

**DOI:** 10.1590/2317-1782/20232022209pt

**Authors:** Maxsuel Alves Avelino de Paiva, Liliane dos Santos Machado, Leonardo Wanderley Lopes

**Affiliations:** 1 Universidade Federal da Paraíba - UFPB, João Pessoa (PB), Brasil.

**Keywords:** Voz, Qualidade da Voz, Percepção Auditiva, Treinamento, Simulação

## Abstract

**Objetivo:**

identificar um conjunto de requisitos para o desenvolvimento de um simulador de treinamento perceptivo-auditivo (TPA) a partir da experiência de docentes que realizam o TPA.

**Método:**

Trata-se de um estudo transversal, descritivo, com abordagem quantitativa. Vinte e dois docentes responderam um questionário *online* contendo 31 itens relacionados ao TPA, envolvendo itens sobre o perfil profissional, condições para o TPA nos cursos de graduação e pós-graduação em Fonoaudiologia, estrutura do TPA, avaliação do efeito do TPA.

**Resultado:**

observou-se que existe variação nos procedimentos de TPA realizados no Brasil. Os principais requisitos indicados pelos respondentes para o TPA envolvem o uso de vozes sintetizadas nos momentos iniciais, seguindo para vozes humanas posteriormente; a utilização de tarefas de fala com vogais sustentadas e fala encadeada; a inserção de informações complementares tais como o gênero, idade, profissão do falante e a espectrografia do sinal vocal; treinamento com tempo mínimo de seis horas; a avaliação do efeito do treinamento pela comparação da concordância intra e inter-juizes pré e pós treinamento; a adição dos parâmetros de grau geral de desvio vocal, rugosidade, soprosidade e tensão; a utilização de escalas contínuas e numéricas validadas; e ser realizado a partir do segundo ano de graduação.

**Conclusão:**

embora haja uma variabilidade da resposta dos especialistas, foi identificado um conjunto mínimo de requisitos indicados para a realização de TPA com novos juízes.

## INTRODUÇÃO

A análise perceptivo-auditiva (APA) da voz é considerada o padrão-ouro entre os métodos utilizados pelo fonoaudiólogo para a avaliação clínica da voz. Ela consiste em um julgamento, a partir das impressões auditivas (percepção) do avaliador, acerca das características vocais do falante, tais como a qualidade vocal, o *pitch*, a *loudness*, a ressonância, a articulação, entre outros^(1)^. A APA permite identificar a presença/ausência de um desvio vocal, caracterizar a intensidade e o tipo de desvio presente na voz, além de fornecer informações importantes quanto à aceitabilidade social dessa voz. Por sua natureza impressionística, a APA é considerada subjetiva e sofre influência de diversos fatores, entre eles, o treinamento do juiz^(2)^. A subjetividade e a natureza arbitrária inerentes a esse método de avaliação podem justificar a tendência ao uso da nomenclatura “julgamento perceptivo-auditivo” (JPA) em substituição à expressão “análise perceptivo-auditiva”^(3,4)^.

O treinamento das habilidades relacionadas ao JPA desenvolve mudanças no sistema de percepção e no processamento da informação auditiva, o que melhora a capacidade do ouvinte em responder aos estímulos treinados. Essas mudanças criam uma memória auditiva que é acessada durante futuras avaliações, permitindo o reconhecimento de padrões julgados normais ou desviados^(5)^.

As estratégias como o uso de âncoras auditivas, o *feedback* imediato e o uso de escalas padronizadas são utilizados no treinamento perceptivo-auditivo (TPA) com o objetivo de reduzir a sua subjetividade^(6-9)^. Tais estratégias possuem pontos negativos, tais como as despesas com materiais (papel, caneta, caixa de som, entre outros), a inviabilidade para a análise imediata do efeito do treinamento e a disponibilidade dos juízes para encontros presenciais. Além disso, o TPA é realizado de diversas formas, e isto dificulta a comparação dos requisitos e dos efeitos dos treinamentos^(10)^.

Com o surgimento de novas tecnologias, as estratégias tradicionais de ensino vêm sendo complementadas com abordagens de ensino interativas. O uso de aplicações como simuladores de treinamento (ST) e *serious games* (SG) possibilitam experiências controladas, incluindo diversas situações que o profissional irá encontrar nos cenários reais, o que pode facilitar a aprendizagem e a transferência dessas habilidades para a realidade prática^(11)^. A utilização de ambientes virtuais permite que os erros sejam cometidos e corrigidos desde as fases iniciais do treinamento, sem acarretar prejuízos para pacientes e alunos. Além disso, é possível realizar a avaliação imediata do desempenho do usuário por meio de medidas objetivas resultantes da interação entre o usuário e o ambiente virtual^(11)^.

Nesse sentido, a primeira etapa para o desenvolvimento de uma aplicação para esse tipo de treinamento é a definição da estrutura do treinamento, dos requisitos e dos balizadores que serão utilizados para compor a aplicação e realizar a avaliação de desempenho do usuário^(11)^. Quando essas definições não são bem estabelecidas na literatura e/ou não existe consenso entre os pesquisadores da área^(12)^, isso se torna um desafio para a proposição de um ST. Nesses casos, a combinação da experiência prática de especialistas e o conhecimento relatado na literatura é a principal estratégia para as definições dos requisitos e para a modelagem de um ST robusto e adequado às necessidades reais^(13)^.

Eventualmente, podem ocorrer divergências entre o conhecimento disposto na literatura e o treinamento realizado na prática cotidiana dos especialistas. Além disso, alguns requisitos dispostos na literatura possuem caráter subjetivo, e precisam ser traduzidos de maneira clara e objetiva para a implementação em um ST. Os estudos que abordam o TPA^(10,14)^ não são consistentes quanto à definição do tempo de treinamento realizado; à quantidade de estímulos utilizados; ao número de parâmetros vocais treinados; ao tipo de escala utilizada; à natureza das vozes utilizadas; e, sobretudo, não indicam se existe uma hierarquia para apresentação dos estímulos e tarefas durante o TPA (tais como o treinamento de presença/ausência de desvio vocal, treinamento de predomínio de desvio vocal e treinamento de graus de desvio vocal apresentados de maneira sequencial, por exemplo). Com isso, justifica-se a consulta a docentes especialistas em Voz que possuam experiência com TPA, com o objetivo de estabelecer requisitos que preencham estas lacunas e respaldem as definições adotadas para a modelagem de um ST para o TPA.

O estabelecimento de requisitos para o TPA pode contribuir para o desenvolvimento de um ST para esta avaliação. Uma modalidade de simulador de TPA pode contribuir para a estruturação e padronização do treinamento, permitir a análise do desempenho e a compreensão da curva de aprendizagem dos juízes, flexibilizar o tempo de treinamento. Além disso, pode ser realizado em outros ambientes além das instituições, tendo em vista que não há necessidade de encontros presenciais para a aplicação do treinamento com o simulador. Dessa forma, o objetivo desta pesquisa é identificar um conjunto de requisitos para o desenvolvimento de um simulador de treinamento perceptivo-auditivo (TPA) a partir da experiência de docentes que realizam o TPA.

## MÉTODO

Trata-se de um estudo transversal, descritivo, com abordagem quantitativa. Foi aprovado pelo CEP da Instituição de origem sob parecer nº 4.746.039 e realizado no período de abril a julho de 2021.

Inicialmente, foram consultadas revisões publicadas recentemente sobre o JPA e a realização do TPA^(10,14)^. Dessa forma, foram observadas inconsistências nos processos de treinamento que envolvem as variáveis tempo de treinamento realizado; quantidade de estímulos utilizados; número de parâmetros vocais treinados; tipo de escala utilizada; e natureza das vozes utilizadas. Posto isso, a consulta a especialistas em Voz que realizam o TPA tem o objetivo de reduzir essas inconsistências e basear as definições adotadas para a modelagem de um ST para o TPA.

O questionário utilizado nesta pesquisa foi elaborado em três etapas, a fim de garantir um instrumento que possibilitasse o suporte à definição dos requisitos para o desenvolvimento de um ST para o TPA, a saber: consulta à literatura; opinião de especialistas no desenvolvimento de ferramentas de simulação; e opinião de especialistas em TPA. Na primeira etapa para elaboração do questionário, foi realizado um levantamento na literatura disponível para identificar as principais variáveis a serem consideradas no TPA^(10,14)^. As variáveis adicionadas nesta etapa foram: informações associadas durante o TPA; nº de horas; tipos de vozes; quantidade de vozes; tipo de tarefa de fala; escalas utilizadas; e parâmetros treinados.

Na segunda etapa, as variáveis selecionadas a partir da consulta à literatura foram apresentadas a um fonoaudiólogo especialista em Voz e uma cientista da computação, ambos com experiência no desenvolvimento de ferramentas de simulação para treinamento de habilidades em profissionais da saúde. Nesta etapa, as perguntas foram estruturadas seguindo o modelo do questionário desenvolvido por Macedo e Machado (2015), que pesquisaram, junto a docentes, sobre os requisitos para o treinamento de profissionais da saúde quanto à aplicação de medicamentos injetáveis. O modelo do questionário utilizado foi adaptado devido à falta de instrumentos na área de Voz que tenham por objetivo entender os requisitos para o TPA. Assim, na referida publicação os autores formalizaram uma proposta de roteiro para o desenvolvimento de instrumentos utilizados para obtenção de informações com especialistas para a definição de requisitos a serem utilizados em simuladores.

O processo de desenvolvimento e implementação de um ST requer a atuação profissional interdisciplinar, a fim de produzir ferramentas efetivas e eficientes. Os profissionais das Ciências da Computação atuam na arte, entretenimento, inteligência artificial e programação do ST. Dessa forma, os cientistas da computação integram a equipe interdisciplinar no desenvolvimento de diferentes aplicações, e trabalham associado aos profissionais com *expertise* na área objeto do determinado produto^(11)^. Assim, a participação de uma cientista da computação neste projeto foi essencial para a programação do ST, implementação da inteligência artificial, e design gráfico do ST. Na concepção do questionário, a participação da cientista da computação esteve relacionada a estruturar as perguntas de modo que as variáveis relevantes para serem selecionadas fossem contempladas no questionário e a adicionar outras variáveis consideradas importantes para a modelagem de um ST, como: ano/período de aplicação do TPA; pré-requisitos para iniciar o TPA; descrição auditiva dos parâmetros treinados; hierarquia das etapas do TPA; uso de outras aplicações (jogos ou simuladores) para o TPA; e forma de avaliação do efeito do TPA.

Na terceira etapa, participaram três fonoaudiólogos especialistas em Voz com *expertise* em TPA, seguindo uma amostra bola de neve (um especialista recrutado indicou outro especialista). O primeiro fonoaudiólogo recrutado atua na mesma instituição de origem, pertence ao mesmo grupo de pesquisa dos autores, porém não participou das etapas anteriores. O segundo fonoaudiólogo recrutado atua na mesma instituição de origem dos pesquisadores e é externo ao grupo de pesquisa. O terceiro fonoaudiólogo recrutado é externo à instituição de origem dos autores. Nesta etapa, a expertise dos especialistas foi avaliada segundo a adaptação do sistema de pontuação “*The Fehring Model*” para a área de Voz^(15)^ (TPA), na qual os três especialistas obtiveram pontuação máxima.

De forma individual e remota (por videochamada), a versão do questionário resultado da segunda etapa, composta por 31 itens, foi apresentada aos três fonoaudiólogos com o objetivo de identificar e modificar os itens que não estivessem alinhados quanto a intenção de coleta do item e a interpretação do respondente. Após a leitura de cada item se perguntava o que o especialista compreendeu. Caso a interpretação do especialista estivesse desalinhada com a intenção de coleta do item, o item deveria ser reformulado a partir das sugestões que o especialista fornecesse combinado com as dos demais participantes. Nesta etapa não houve desalinhamento entre as interpretações dos especialistas e o questionário, porém algumas sugestões foram dadas para melhorar a qualidade do instrumento. As modificações realizadas nesta etapa foram: ajustes de texto como no item “Você usa outros recursos durante o treinamento perceptivo-auditivo?” para “Durante o treinamento perceptivo-auditivo você associa alguma outra informação sobre a voz apresentada?”; mudanças no tipo de coleta de respostas como ao invés de usar caixa de seleção “humanas” e “sintetizadas” para o item “As vozes utilizadas no treinamento são:” para múltipla escolha adicionando a opção “ambas”, para facilitar a análise posterior; e redução do número de itens com respostas abertas, para diminuir o tempo de aplicação do questionário e facilitar a participação dos docentes com um instrumento mais prático de responder como no item “Que tarefa de fala você utiliza para o treinamento perceptivo-auditivo com o CAPE-V?”.

A versão final do questionário incluiu 31 itens (5 subjetivos e 26 objetivos) organizados em quatro blocos: 1) Perfil Profissional; 2) Condições para o TPA nos cursos de graduação e pós-graduação em Fonoaudiologia; 3) Estrutura do TPA; e 4) Avaliação do efeito do TPA. A divisão em blocos teve o intuito de tornar compreensível a temática específica da qual os itens se remetem e facilitar a análise e discussão dos resultados. Esta divisão foi feita pelos autores e aprovada pelos fonoaudiólogos especialistas.

Para recrutamento dos participantes, foi enviado *e-mail* para a coordenação de 81 cursos de graduação em Fonoaudiologia e cinco cursos de pós-graduação *lato sensu* em Voz, solicitando-se o contato do professor responsável pelo TPA dos alunos de graduação em Fonoaudiologia ou fonoaudiólogos pós-graduandos, e/ou a divulgação desta pesquisa para esses professores. O *e-mail* continha uma breve descrição da pesquisa, seus objetivos, os critérios para participação e o *link* para acesso ao Termo de Consentimento Livre e Esclarecido (TCLE).

Considerando-se o objetivo do estudo, foram estabelecidos os seguintes critérios de elegibilidade para participação nesta pesquisa: ser docente em nível de graduação e/ou pós-graduação em Fonoaudiologia; ter experiência no ensino de alguma disciplina em que se realize o TPA; e já ter realizado o TPA ao menos uma vez. Os participantes que se enquadrassem nos critérios elencados e estivessem de acordo com o TCLE eram convidados a dar continuidade, respondendo a o questionário na ferramenta *Google Forms.*

Dessa forma, a amostra final foi composta por 22 docentes. Desses, 15 (68,2%) atuavam no nível de graduação e pós-graduação *stricto sensu*, dois (9,1%) atuavam somente no nível da pós-graduação *lato sensu* em Voz e cinco (22,7%) atuavam no treinamento de alunos de graduação e pós-graduação *lato sensu* em Voz. Considerando que a população de docentes que realizem o TPA seja de um docente por instituição, a amostra compõe cerca de 26% da população. Ressalta-se que apesar dos esforços para buscar a participação de mais docentes, muitos *e-mails* não foram respondidos, mesmo após três envios.

A amostra incluiu representantes de três regiões do Brasil (Nordeste, Sudeste e Centro-Oeste) das seguintes unidades da federação: São Paulo (n=10, 45,5%), Pernambuco (n=3, 13,6%), Paraíba (n=3, 13,6%), Minas Gerais (n=2, 9,1%), Rio de Janeiro (n=2, 9,1%), Distrito Federal (n=1, 4,5%) e Rio Grande do Norte (n=1, 4,5%). De acordo com o perfil profissional, os entrevistados foram, em sua maioria (n=13, 59,1%), doutores com mais de 10 anos de docência em cursos de graduação em instituições públicas, onde realizam o TPA em componentes curriculares obrigatórios.

A planilha de dados foi extraída do *Google Forms* e foram calculadas medidas de frequência relativa para os itens com respostas objetivas. Os itens com respostas subjetivas foram analisados de forma qualitativa e agrupados em categorias, de acordo com o que as respostas mencionavam.

## RESULTADOS

Sobre as condições para o treinamento nos cursos de graduação em Fonoaudiologia, a maior parte dos entrevistados (n=21, 95,5%) afirma que o TPA é feito a partir do segundo ano do curso de graduação. Quando perguntados se há um melhor momento ao longo da graduação para que este treinamento seja realizado, 15 dos entrevistados (68,18%) afirmaram que existe e todos concordam que deve ser realizado a partir do segundo ano do curso de graduação (Quadro 1).

**Quadro 1:** Itens do questionário e respostas dos participantes

BLOCO 1 - PERFIL PROFISSIONAL		
ITENS		***RESPOSTA DOS ENTREVISTADOS***
1	Qual o seu grau máximo de formação?	*Doutorado (n=13, 59,1%)*
		*Pós-doutorado (n=9, 40,9%)*
2*	Para qual público você desenvolve/aplica treinamento perceptivo-auditivo da Voz?	*Graduação (n=19, 86,4%)*
		*Especialização em Voz (n=7, 31,8%)*
		*Pós-graduação Stricto Sensu (n=9, 40,9%)*
		*Cursos livre (n=5, 22,7%)*
		*Residência (n=1, 4,5%)*
3	Se leciona na graduação, o treinamento perceptivo-auditivo para avaliação da qualidade vocal faz parte do conteúdo de algum componente curricular obrigatório?	*Sim (n=15,68,2% )*
		*Não (n=1, 4,5%)*
		*NA (n=6, 27,3%)*
4*	Tipo de instituição em que ensina?	*Pública (n=18, 81,9%)*
		*Particular (n=4, 18,2%)*
		*Fundação (n=1, 4,5%)*
5	UF da instituição?	*São Paulo (n=10, 45,5%), Paraíba (n=3, 13,6%), Pernambuco (n=3, 13,6%), Minas Gerais (n=2, 9,1%), Rio de Janeiro (n=2, 9,1%), Distrito Federal (n=1, 9,1%) e Rio Grande do Norte (n=1, 9,1%)*
6	Tempo de docência em anos?	*Mais de 10 anos (n=18, 81,1%)*
		*Menos de 10 anos (n=4, 18,2%)*
BLOCO 2) CONDIÇÕES PARA O TREINAMENTO PERCEPTIVO-AUDITIVO		
ITENS		***RESPOSTA DOS ENTREVISTADOS***
1	Em que ano do curso de Graduação os alunos recebem o treinamento perceptivo-auditivo na instituição que você atua?	*A partir do 2º ano (n=21, 95,5%)*
2	Você considera que há um melhor momento do curso ao longo da graduação para oferecer um programa de treinamento perceptivo-auditivo para os estudantes de Fonoaudiologia?	*Sim (n=15, 68,2%)*
		*Todos afirmam que deve ser realizado a partir do segundo ano da graduação*
		*Não (n=7, 31,8%)*
3	Existe algum pré-requisito para os alunos iniciarem este treinamento na instituição que você atua?	*Sim (n=10, 45,5%)*
		*Não (n=12, 54,5%)*
BLOCO 3) ESTRUTURA DO TREINAMENTO PERCEPTIVO-AUDITIVO		
ITENS		***RESPOSTA DOS ENTREVISTADOS***
1*	Durante o treinamento perceptivo-auditivo você associa alguma outra informação sobre a voz apresentada?	*Idade (n=18, 81,1%)*
		*Gênero (n=18, 81,1%)*
		*Queixa principal (n=14, 63,6%)*
		*Espectrograma (n=11, 50%)*
		*Exame laríngeo (n=10, 45,4%)*
2	Qual o total de horas de treinamento em sala de aula?	*Menos de 2h (n=2, 9,1%)*
		*Entre 2 e 4h (n=5, 22,7%)*
		*Entre 4 e 6h (n=3, 13,6%)*
		*Entre 6 e 8h (n=5, 22,7%)*
		*Mais de 8h (n=4, 18,1%)*
		*Outros (n=3, 13,6%)*
3	As vozes utilizadas no treinamento são humanas e/ou sintetizadas?	*Humanas (n=17, 77,3%)*
		*Humanas e Sintetizadas (n=5, 22,7%)*
4	Quantidade de vozes que você utiliza no treinamento?	*Até 20 vozes (n=8, 36,4%)*
		*Entre 21 e 40 vozes (n=7, 31,8%)*
		*Mais de 50 vozes (n=7, 31,8%)*
5*	Que tarefas de fala você utiliza para o treinamento?	*Contagem de números (n=21, 95,5%)*
		*Frases do CAPE-V (n=17, 77,3%)*
		*Fala espontânea (n=17, 77,3%)*
		*Vogal “a” (n=15, 68,2%)*
		*Vogal “é” (n=12, 54,5%)*
6	Você utiliza o instrumento CAPE-V (Kempster et al.^(16)^) no treinamento perceptivo-auditivo?	*Sim (n=18, 81,8%)*
		*Não (n=4, 18,2%)*
7	Você utiliza o instrumento GRBAS (Hirano^(17)^) no treinamento perceptivo-auditivo?	*Sim (n=20, 90,9%)*
		*Não (n=2, 9,1%)*
8	Você utiliza a Escala de Desvio Vocal - EDV (Yamasaki et al.^(18)^) no treinamento perceptivo-auditivo?	*Sim (n=13, 59,1%)*
		*Não (n=9, 40,9%)*
9**	Suponha que existam 3 níveis hierárquicos de complexidade para o treinamento perceptivo-auditivo. Indique a ordem dos níveis que você utilizaria no treinamento?	*1º Nível: Identificação da presença do desvio vocal*
		*(n=20, 90,9%)*
		*2º Nível: Avaliação da qualidade vocal predominante*
		*(n=15, 68,2%)*
		*3º Nível: Avaliação do grau geral do desvio vocal (n=12, 54,5%)*
10*	Quais parâmetros você inclui no treinamento?	*Rugosidade (n=22, 100%)*
		*Soprosidade (n=22, 100%)*
		*Tensão (n=22, 100%)*
11^#^	Como você define para os alunos as características auditivas que são esperadas para uma voz rugosa?	*Respostas não específicas ao definir características auditivas ou definem correlatos anatomofisiológicos:*
		*“Irregularidade vibratória”, “ruído”, “voz suja”, “presença de areia”*
12^#^	Como você define para os alunos as características auditivas que são esperadas para uma voz soprosa?	*Respostas faziam referência a:*
		*“Qualquer escape de ar audível durante a produção vocal”*
13^#^	Como você define para os alunos as características auditivas que são esperadas para uma voz tensa?	*Respostas não específicas ao definir características auditivas ou definem correlatos anatomofisiológicos:*
		*“Sensação de aperto”, “esforço vocal”, “hiperfunção vocal”*
14	Quantas horas de treinamento você considera que seriam suficientes para melhorar o desempenho (taxa de acertos) e a confiabilidade dos alunos na análise perceptivo-auditiva?	*Até 4 horas (n=3, 13,6%)*
		*Entre 5 e 8 horas (n=5, 22,7%)*
		*Mais de 8 horas (n=14, 63,3%)*
15	Você utiliza algum tipo de jogo ou simulador para treinamento perceptivo-auditivo?	*Sim (n=1, 4,5%)*
		*Não (n=21, 95,5%)*
BLOCO 4) AVALIAÇÃO DO EFEITO DO TREINAMENTO PERCEPTIVO-AUDITIVO		
ITENS		***RESPOSTA DOS ENTREVISTADOS***
1	*Você faz a avaliação da confiabilidade dos alunos na análise perceptivo-auditiva após o treinamento perceptivo-auditivo?*	Sim (n=9, 40,9%)
		Não (n=13, 59,1%)
2^#^	*Como você avalia o efeito do treinamento perceptivo-auditivo sobre o desempenho e a confiabilidade do aluno?*	As respostas citam: “Teste de concordância inter e intra-avaliador; observação e discussão das avaliações; Prova formal”
3	*Você tem dificuldade para avaliar o efeito do treinamento perceptivo-auditivo? (Caso não faça a avaliação do efeito do treinamento, marque "NA")*	Sim (n=5, 22,7%)
		Não (n=6, 27,3%)
		Não avaliam (n=11, 50%)
4#	*Pode referir alguma(s) dificuldade(s) para avaliar o efeito do treinamento perceptivo-auditivo?*	As dificuldades envolvem: “Falta de parâmetro para comparação; Falta de instrumentos calibradores para treino; Procedimento trabalhoso com teste estatístico”
5	*Como você define o "acerto" do aluno quanto ao grau geral do desvio vocal usando o CAPE-V ou a EDV?*	Quando a marcação do CAPE-V ou EDV coincide com os valores do juiz de referência ou está 10 mm para mais ou para menos desse valor (n=13, 59,1%)
		*Quando a marcação do CAPE-V ou EDV coincide com os valores do juiz de referência ou está 5 mm para mais ou para menos desse valor (n=2, 9,1%)*
		*Quando a marcação do CAPE-V ou EDV coincide com os valores do juiz de referência, sem margens de erro (n=2, 9,1%)*
		*Não utilizo o CAPE-V ou EDV (n=4, 18,2%)*
		*Outro: (n=1, 4,5%)”Nunca utilizei esses critérios”*
6	*Como você avalia o acerto do aluno na avaliação do grau geral, caso use a escala GRBAS?*	Quando coincide (n=10, 45,5%)
		Quando coincide ou está um grau para mais ou para menos (n=10, 45,5%)
		Não utilizo a GRBAS (n=2, 9%)
7	*O quanto considera que o treinamento perceptivo-auditivo é importante na formação inicial do aluno de Fonoaudiologia em uma escala de 0 - (não é importante) a 5 - (Muito importante)?*	5 (n=19, 86,4%)
		4 (n=2, 9,1%)
		3 (n=1, 4,5%)

**Legenda:** * Item que permite a escolha de mais de uma resposta; ** Neste item são apresentados os níveis mais citados como 1º, 2º e 3º nível do TPA; ^#^ Itens subjetivos que permitiam os docentes formularem suas respostas

De maneira geral, os respondentes associam outras informações dos falantes durante o TPA, tais como o gênero (n=18, 81,8%), a idade (n=18, 81,8%), a queixa (n=14, 63,3%), o espectrograma (n=11, 50%) e o resultado do exame laríngeo (n=10, 45,5%) dos falantes.

O número total de horas de TPA em sala de aula variou de menos de 2hs a mais de 8hs. A maior parte das respostas estão em um intervalo entre 6 e 8 horas de treinamento (n=5, 22,73%).

A maioria dos participantes utilizam vozes humanas (n=17, 77,3%) na realização do TPA, enquanto cinco (22,7%) utilizam vozes humanas e sintetizadas. A quantidade de vozes utilizadas no TPA foi semelhante entre os intervalos “Até 20 vozes” (n=8, 36,4%), “Entre 21 e 40 vozes” (n=7, 31,8%) e “Mais de 50 vozes” (n=7, 31,8%).

As tarefas de fala mais utilizadas no TPA são contagem de números (n=21, 95,5), fala espontânea (n=17, 77,3%), frases do CAPE-V (n=17, 77,3%), vogais /a/ (n=15, 68,2%) e vogal /ɛ/ (n=12, 54,5%). As escalas mais usadas são a GRBAS (n=20, 90,9%) e a CAPE-V (n=18, 81,8%).

De modo geral, os respondentes iniciam o TPA pela identificação da presença/ausência de desvio vocal. Porém, divergem quanto à sequência das etapas posteriores. Quando perguntados sobre uma possível hierarquização das etapas do TPA, a maioria indicou a seguinte ordem de aplicação: identificação da presença de desvio vocal como 1º nível de treinamento (n=20, 90%), avaliação da qualidade vocal predominante como 2º nível (n=15, 68,2%) e avaliação do grau de desvio vocal como último nível (n=12, 54,5%).

Todos os entrevistados incluem os parâmetros grau geral de desvio vocal (G), rugosidade (R), soprosidade (B) e tensão (S) no TPA. Quando solicitada a descrição de características auditivas relacionadas aos parâmetros incluídos no treinamento, os entrevistados parecem demonstrar dificuldades para definir características auditivas para os parâmetros R e S. O parâmetro B, do ponto de vista auditivo, parece ser mais fácil de ser explicado. As respostas dos entrevistados se referiam à: “Qualquer escape de ar audível durante a produção vocal”. As respostas dos entrevistados não foram específicas ao definir características auditivas e/ou definiam os correlatos anatomofisiológicos para R e S. Para R, as respostas se referiam à: “irregularidade vibratória”, “ruído”, “voz suja”, “presença de areia”. Para S, as respostas faziam referência à: “Sensação de aperto”, “esforço vocal”, “hiperfunção vocal”.

Mais da metade dos entrevistados (n=14, 63,3%) considera que o TPA deve ter duração acima de 8 horas para alcançar melhoria no desempenho e na confiabilidade dos juízes. A maioria dos participantes (n=21, 95,5%) não utiliza nenhum tipo de simulador ou jogo para o TPA.

Cerca de 60% dos entrevistados (n=13) não fazem avaliação da confiabilidade dos juízes após o TPA. Dentre os que avaliam a confiabilidade após o TPA, os métodos citados foram: teste de concordância inter e intra-avaliador; observação e discussão das avaliações; Prova (avaliação formal). Desses, 22,7% (n=5) referem ter dificuldade para a avaliação do efeito do TPA pela falta de parâmetros para comparação, falta de instrumentos calibradores para treino e porque o procedimento com teste estatístico é inviável de ser realizado durante o TPA.

Mais da metade dos entrevistados (n=13, 59,1%) que utilizam os instrumentos CAPE-V e a Escala de Desvio Vocal (EDV) considera como correta a avaliação que coincide com os valores do juiz de referência ou está 10 mm para mais ou para menos desse valor. Entre os entrevistados que utilizam a escala GRBAS, 50% (n=10) considera como correta a avaliação que coincide com o grau atribuído pelo juiz de referência e a outra parte (n=10, 50%) admite um grau para mais ou para menos. O TPA é considerado muito importante na formação inicial do aluno de Fonoaudiologia para 86,4% (n=19) dos entrevistados.

## DISCUSSÃO

O JPA é influenciado por diversos fatores, entre eles, o treinamento dos juízes^(2)^. Os modelos de TPA registrados na literatura apresentam inconsistências a respeito das variáveis envolvidas no treinamento^(10,14)^. Para definir a melhor forma de realizar o TPA é necessário, inicialmente, conhecer os diversos métodos de treinamento já utilizados e registrados na literatura. Além disso, estes modelos devem ser descritos de modo que permitam a avaliação adequada dos seus resultados, a comparação entre treinamentos, e a determinação para quais populações os resultados podem ser aplicáveis^(10)^.

Estabelecer requisitos para que um treinamento seja realizado por meio de um ST não é uma tarefa fácil, sobretudo, quando se trata de um treinamento subjetivo como o TPA. Para suprir estas dificuldades e tornar as definições dos requisitos do ST mais objetivas, a combinação entre o conhecimento disponível na literatura e a experiência prática de especialistas torna-se essencial para a proposição de um ST mais robusto^(13)^.

Dessa forma, a consulta a juízes com expertise em TPA no planejamento do treinamento de novos fonoaudiólogos é fundamental para o desenvolvimento de modelos de treinamento baseado na opinião de especialistas^(11)^. Juízes experientes possuem padrões internos mais definidos e experiência para conduzir o treinamento de iniciantes. Os padrões internos são gerados pelo TPA e pelas experiências com o JPA ao longo da sua formação acadêmica e do exercício profissional^(12)^. Dessa forma, tanto a formação profissional (treinamento formal durante a graduação e pós-graduação) quanto a característica temporal (anos de atuação com JPA) têm sido referidas para determinar a experiência de um juiz^(14)^. Neste estudo, ambas as formas foram consideradas, tendo em vista que todos os entrevistados foram fonoaudiólogos doutores, com mais de 10 anos de docência e com experiência em JPA e TPA.

Nesta pesquisa, observou-se que os especialistas concordam que o TPA deve ser realizado a partir do segundo ano do curso de graduação. Nos anos iniciais da graduação, os alunos estudam disciplinas básicas das ciências da saúde que são necessárias para entender o comportamento anatomofisiológico correspondente à qualidade vocal das vozes avaliadas.

A classificação do desvio vocal depende de algumas informações complementares à voz como o gênero, a idade e a profissão do falante. Além disso, o apoio visual do traçado espectrográfico pode aumentar significativamente a confiabilidade do JPA da qualidade vocal de juízes inexperientes, porque promove aumento da concordância inter e intra juízes da maior parte dos parâmetros analisados^(19)^. Sendo assim, justifica-se a adição destas informações junto à apresentação das vozes durante o treinamento de juízes inexperientes.

Apesar de a maioria dos entrevistados utilizarem vozes humanas para o TPA, o uso de vozes sintetizadas parece ser mais adequado para esta finalidade, principalmente durante os momentos iniciais do treinamento de juízes inexperientes^(14,20)^. Em vozes sintetizadas, os parâmetros vocais podem ser controlados para a produção de vozes unidimensionais (apenas um parâmetro desviado) e, assim, simplificar a avaliação de juízes inexperientes^(20)^.

A quantidade de vozes utilizadas parece não ser explorada nos estudos da área como uma variável importante para o TPA. Essa quantidade possui muita variação e alguns estudos mencionam a utilização de 30^(8)^, 57^(9)^, e 220^(21)^ vozes. Considerando um TPA que envolva os parâmetros mais universais (R, B e S), os diversos graus (leve, moderado e intenso) e o pareamento por gênero (masculino e feminino), entende-se que o intervalo de 30 a 60 vozes é minimamente suficiente para a realização do TPA.

A utilização de tarefas de fala como vogais sustentadas e fala encadeada permitem a avaliação tanto de informações correspondentes a fonte glótica quanto aos ajustes musculares realizados no trato vocal^(14)^. Associações entre tarefa de fala, ajustes musculares e correlatos auditivos são importantes para o treinamento de juízes iniciantes e por isso justifica-se a variedade de tarefas vocais durante o TPA.

A quantidade de horas e o número de estímulos utilizado no TPA apresentaram uma ampla variação entre as respostas dos entrevistados. Estas variações também são encontradas na revisão sobre métodos de TPA de Walden e Khayumov (2020), em que o tempo de treinamento variou de 30 minutos a 20 horas^(10)^. Um estudo^(22)^ que utilizou estímulos âncoras durante o TPA, observou aumento da confiabilidade intra e interavaliadores após duas horas de treinamento. Tendo em vista a opinião dos entrevistados e os estudos na área, espera-se que haja aumento na confiabilidade dos juízes a partir de seis a oito horas de treino. A definição mais precisa sobre o tempo necessário para TPA só será possível com estudos que avaliem o desempenho dos juízes em diferentes intervalos de tempo durante o TPA.

Quanto aos instrumentos utilizados para a marcação do JPA, a escala GRBAS e o protocolo CAPE-V são os mais utilizados e aceitos mundialmente no contexto clínico e científico. Estes instrumentos ajudam na padronização da APA e possuem particularidades quanto ao modo de marcação, aos parâmetros que avaliam e ao tipo de tarefa de fala a ser utilizada^(14)^. Não se sabe qual o melhor instrumento a ser utilizado no treinamento de fonoaudiólogos em formação, por isso, recomenda-se que a escolha do instrumento seja baseada no objetivo do treinamento, nas amostras de falas disponíveis e no tempo de treinamento estimado^(14)^.

Os descritores G, R, B e S estão entre os parâmetros universais mais utilizados no JPA^(14)^. Destes, os que apresentam maiores concordâncias são o G, o R e o B. O parâmetro S apresenta menor concordância inter e intra juízes, sendo considerado menos confiável no JPA em comparação aos outros parâmetros^(14,23,24)^. Todos os entrevistados incluem os parâmetros de G, R, B e S na aplicação do TPA. Portanto, justifica-se a inclusão, minimamente, destes parâmetros no treinamento da APA.

A descrição de características propriamente auditivas para os parâmetros de R e S são um desafio para os entrevistados. Apesar do parâmetro R ser recorrente na avaliação clínica da voz, ao tentar descrever características auditivas, os entrevistados utilizam a descrição de correlatos anatomofisiológicos que ocorrem no momento da fonação. O mesmo ocorre para o parâmetro S, no qual confundem-se os conceitos de esforço (percepção vivenciada pelo falante) e tensão (atividade de contratilidade muscular)^(25)^.

Para a sequencialização dos níveis do TPA, entende-se que a identificação da presença/ausência do desvio vocal seja mais fácil para juízes iniciantes por se tratar de uma classificação categórica e binária. A caracterização do tipo de desvio da qualidade vocal predominante exige uma classificação categórica mais complexa, incluindo, no mínimo, três possibilidades (rugosa, soprosa ou tensa). Entende-se que o nível mais complexo do TPA seja a avaliação da intensidade do desvio vocal, seja para o desvio global ou para a avaliação dos componentes de rugosidade, soprosidade e tensão. Neste nível, a avaliação é baseada em um *continuum* que vai desde a ausência de uma determinada característica vocal até a presença em grau intenso desta característica.

Uma forma de avaliar o efeito do TPA é a utilização de medidas de concordância intra e interavaliador. Os testes estatísticos de concordância quantificam a proximidade das avaliações antes e depois do TPA^(26)^. Espera-se que haja maior proximidade entre as avaliações dos juízes inexperientes e o juiz de referência após o TPA (concordância interavaliador). Também é esperado que os juízes inexperientes se tornem mais consistentes em suas avaliações (concordância intra-avaliador). Por isso, é importante a realização da avaliação do efeito do TPA para monitoramento dos resultados obtidos e implementação de novas estratégias que sejam necessárias.

Quando utilizado instrumentos com escalas contínuas de 100 mm para o JPA, tais como contínuos CAPE-V e EDV, admite-se uma variabilidade de até 10 mm entre avaliadores, desde que tal variação não ultrapasse os limites (valores de corte) para modificar o grau do desvio avaliado^(27)^.

## CONCLUSÃO

Existe variação nas respostas dos entrevistados quanto aos procedimentos de TPA. A partir da pesquisa com docentes experientes em JPA e o conhecimento disposto na literatura, o desenvolvimento de um ST para o TPA deve considerar os seguintes requisitos: iniciar o TPA pela tarefa de classificação de presença/ausência de desvio vocal, avançando para a classificação da predominância da qualidade vocal e, por último, a classificação dos graus de desvios vocais; usar de vozes sintetizadas nos momentos iniciais seguindo para vozes humanas posteriormente; utilizar entre 30 a 60 vozes; utilizar tarefas de fala com vogais sustentadas e fala encadeada; inserir informações complementares como o gênero, idade, profissão do falante e espectrografia da voz; conter no mínimo 6 horas de treinamento; avaliar o efeito do treinamento pela comparação da concordância intra e inter-juiz pré e pós treinamento; adicionar os parâmetros de grau geral de desvio vocal, rugosidade, soprosidade e tensão (no mínimo); utilizar escalas contínuas e numéricas; e ser realizado a partir do segundo ano de graduação.

Destaca-se que os requisitos estabelecidos são flexíveis e podem ser alterados com o avançar dos estudos na área, porém servem como ponto de partida para a proposição e o desenvolvimento de um ST.
